# Case of Melancholia, from a Material Cause in the Brain, Terminating in Effusion of Blood There, and Apoplexy; with the Appearances on Dissection

**Published:** 1816-10

**Authors:** James Foy

**Affiliations:** Surgeon in His Majesty's Naval Service


### JOURNAL AND REVIEW.

VOL. II.] OCTOBER, 1816. [NO. 10.

### PART I.

### ORIGINAL COMMUNICATIONS.

—— quid utile ——

*Case of Melancholia, from a material Cause in the Brain, terminating in Effusion of Blood there, and Apoplexy; with the Appearances on Dissection.* By JAMES FOY, Surgeon in His Majesty's Naval Service.

THE various shades of Hypochondriasis and Melancholia, however distressing, or even dangerous to the patient, excite comparatively little commiseration in the friends or the physician; probably, from the belief that the sufferings are only ideal, or the effects of a disordered fancy, and therefore only to be combated by moral means. But Mania, which is perhaps only the superlative degree of these affections, is now I believe pretty generally found to spring from, or at least, to be accompanied by, *material* disorganization in some viscus, particularly the brain. Perhaps the following sketch may be deemed worthy of a place in your valuable Journal.

Mr. ——— L——d, a gentleman, about 47 years of age, and naturally of a melancholic temperament, was observed, in the early part of November 1815, to labour under very considerable dejection of spirits, accompanied by an apparent aversion to society and conversation, and a desire for solitude, alleging as a reason, that the state of his health did not admit of conviviality. He complained of great debility; said he was feverish, and could not sleep at night. He had very little appetite for food, but had much thirst; complained that the lower extremities felt benumbed, and powerless. As he had suffered a paralytic affection about two years before, it was apprehended that the present symptoms were prelusive of a second attack of that disease; but a short space of time changed considerably the scene, or at least the apparent source of his uneasiness. His bodily health was now no longer complained of; but he conceived that his affairs were in so deranged a state, that immediate ruin must be the consequence. These imaginary evils drove him to the greatest despair; he sought opportunities of destroying himself, and was twice frustrated in the suicidal attempts! Notwithstanding all possible means were tried to alleviate his misery, he still laboured under such incessant and excessive grief, bewailing his misfortunes, bewailing even his existence, and extracting sorrow from every reflection, that it was painful and distressing to see him! His memory was, all this time, extremely tenacious, particularly of events long past: His appetite was much better than before his mental illness, and his judgement, when not exercised on his own affairs, appeared to be tolerably correct. His eyes had often a watery appearance, and, at times, he would glance suddenly at particular persons, and assume a scrutinizing and most expressive look. Great watchfulness and inability to sleep increased his sufferings by night, yet this lamentable mental despondency, and almost total want of rest, affected not his corporeal health; which appeared as good as ever it had been in his life.

Venæsection and cathartics were employed whenever any determination to the head appeared; but as the cause was supposed to be a *mental* one, or some concealed misfortune, our principal efforts were directed towards tranquillizing his mind, subduing his fears, and assuring him that his affairs would be satisfactorily settled.

On the morning of the 18th of June 1816, after having appeared in unusually good spirits the night before, he was found labouring under all the symptoms of apoplexy. He recovered, however, so far from a state of insensibility, as to be able to swallow and make efforts to speak; but the whole of the right side was paralytic. He now vomited some yellowish matters; the fæces passed involuntarily, and he appeared to suffer from strangury. In about ten hours, a second attack followed; he became totally insensible; breathing very stertorous, with slight tremors, and a strong frequent pulse. As there were some reasons to suspect that a poisonous substance had been swallowed, a dose of tartrite of antimony was immediately exhibited, but with little effect. A large quantity of blood was abstracted from the temporal artery; purgatives were given by the mouth; cathartic enemas thrown up. Blisters were applied to the head; frictions to the affected side, &c. &c. but all in vain; he died early on the morning of the 20th of June.

*Sectio cadaveris.* Twelve hours after death.

*Abdomen.* Part of the small intestines, and the inner coat of the stomach near its cardiac orifice were in a high state of inflammation, which appearances strengthened the suspicion of poison having been swallowed. The contents of the stomach were only a small quantity of blackish fluid, from which a dark coloured powder readily subsided. No test that I could apply made any discovery of its nature.

*Head.* The vessels of the brain were very turgid throughout, particularly those on the surface of the cerebellum. Adhesions, by means of a granulated substance, had taken place between the dura and pia mater, near the posterior extremity of the sagittal suture, and near them were several small depositions of bone, in the form of *spicula,* on the *inner* surface of the dura mater. The *immediate* cause, however, of the fatal termination was an extravasation of blood in the substance of the cerebrum over the *left* ventricle, into which part of the blood had forced itself. A considerable quantity of water, with some blood, was also effused into the right ventricle. The quantity of blood extravasated on the left side exceeded four ounces. The cartilages of all the ribs were completely ossified.

I think a sufficient and satisfactory cause may be assigned for the train of symptoms in this case. The formation of spicula of bone on the inner surface of the dura mater must have kept up a constant irritation in the delicate organ underneath; and this may be readily supposed capable of exciting violent and irregular action in the vessels with a consequent determination of blood to the encephalon, at length ending in rupture and extravasation.*

---

* Mr. Foy has favoured us with some of these ossific depositions. They are extremely irregular, and spiculated in their form, and seem capable of producing great irritation in the surrounding soft parts. EDITORS.

The causes of the inflammatory appearances in the stomach and intestines I am unable to explain; for the most rigid investigation did not sanction the suspicion of any noxious substance having been swallowed. The cartilages of the ribs having been all converted into bone, shews the general tendency towards ossific deposition in the arteries of this unfortunate gentleman.

*July* 15*th*, 1816. JAMES FOY.

